# Trends in the prevalence and distribution of HTLV-1 and HTLV-2 infections in Spain

**DOI:** 10.1186/1743-422X-9-71

**Published:** 2012-03-23

**Authors:** Ana Treviño, Antonio Aguilera, Estrella Caballero, Rafael Benito, Patricia Parra, Jose M Eiros, Araceli Hernandez, Enrique Calderón, Manuel Rodríguez, Alvaro Torres, Juan García, Jose Manuel Ramos, Lourdes Roc, Goitzane Marcaida, Carmen Rodríguez, Matilde Trigo, Cesar Gomez, Raul Ortíz de Lejarazu, Carmen de Mendoza, Vincent Soriano

**Affiliations:** 1 Infectious Diseases Department, Hospital Carlos III, Calle Sinesio Delgado 10, Madrid 28029, Spain; 2 Service of Microbiology, Hospital Conxo, Santiago de Compostela, Spain; 3 Service of Microbiology, Hospital Vall d'Hebron, Barcelona, Spain; 4 Service of Microbiology, Hospital Clínico Universitario Lozano Blesa, Zaragoza, Spain; 5 Service of Microbiology, Hospital Clínico Universitario, Valladolid, Spain; 6 Service of Microbiology, Hospital Universitario Insular, Las Palmas de Gran Canaria, Spain; 7 CIBER in Public Health and Epidemiology, Hospital Universitario Virgen del Rocío, Seville, Spain; 8 Service of Microbiology, Hospital Universitario Puerta del Mar, Cádiz, Spain; 9 Service of Microbiology Hospital Universitario de Canarias, Santa Cruz de Tenerife, Spain; 10 Service of Microbiology, Hospital Cristal-Piñor, Orense, Spain; 11 Infectious Disease Unit, Hospital General, Elche, Spain; 12 Service of Microbiology, Hospital Miguel Servet, Zaragoza, Spain; 13 Center of Biomedical Diagnostic, Hospital General Universitario, Valencia, Spain; 14 Centro Sanitario Sandoval, Madrid, Spain; 15 Service of Microbiology, Complejo Hospitalario, Pontevedra, Spain; 16 Service of Microbiology, Complejo Hospitalario Virgen de la Salud, Toledo, Spain

**Keywords:** HTLV, Spain, Seroprevalence, Epidemiology, HTLV-3, HTLV-4

## Abstract

**Background:**

Although most HTLV infections in Spain have been found in native intravenous drug users carrying HTLV-2, the large immigration flows from Latin America and Sub-Saharan Africa in recent years may have changed the prevalence and distribution of HTLV-1 and HTLV-2 infections, and hypothetically open the opportunity for introducing HTLV-3 or HTLV-4 in Spain. To assess the current seroprevalence of HTLV infection in Spain a national multicenter, cross-sectional, study was conducted in June 2009.

**Results:**

A total of 6,460 consecutive outpatients attending 16 hospitals were examined. Overall, 12% were immigrants, and their main origin was Latin America (4.9%), Africa (3.6%) and other European countries (2.8%). Nine individuals were seroreactive for HTLV antibodies (overall prevalence, 0.14%). Evidence of HTLV-1 infection was confirmed by Western blot in 4 subjects (prevalence 0.06%) while HTLV-2 infection was found in 5 (prevalence 0.08%). Infection with HTLV types 1, 2, 3 and 4 was discarded by Western blot and specific PCR assays in another two specimens initially reactive in the enzyme immunoassay. All but one HTLV-1 cases were Latin-Americans while all persons with HTLV-2 infection were native Spaniards.

**Conclusions:**

The overall prevalence of HTLV infections in Spain remains low, with no evidence of HTLV-3 or HTLV-4 infections so far.

## Background

Four different types of human T-lymphotropic viruses (HTLV), named 1-4, have been described in humans. HTLV-1, the first human retrovirus was identified in 1980; it is the etiological agent of adult T-cell leukemia/lymphoma (ATLL) [1] and tropical spastic paraparesis/HTLV-1 associated myelopathy (TSP/HAM) [2]. These illnesses fortunately only affect to less than 10% of infected individuals lifetime. HTLV-2 was identified in 1982; it has occasionally been associated with subacute neurological syndromes resembling TSP/HAM [3] with no evidence of producing hematological malignancies [4]. Finally, HTLV-3 and HTLV-4 were described in 2005 in a few asymptomatic individuals from Cameroon and to date no illnesses have been associated with these viral infections [5-8].

The main routes of transmission of HTLV are from infected mothers to their newborns, especially through prolonged breast-feeding, sexual intercourse, blood transfusion and sharing of needles and syringes between intravenous drug users [9]. HTLV-1 has spread worldwide with estimates of 10-20 million infected people. It is endemic in some parts of Japan, Central and South America and Sub-Saharan Africa [10]. In contrast, HTLV-2 infection affects 3-5 million persons and is prevalent in some Amerindian and African pygmy tribes and epidemic among injecting drug users in Western Europe and North America [4]. In Spain, the majority of individuals HTLV positive are native Spaniards, most of them with past history of intravenous drug use and infected with HTLV-2. In contrast, most persons infected with HTLV-1, which is overall less prevalent, are immigrants coming from endemic regions in Central and South America [11-13]. Until December 2009, a total of 144 cases of HTLV-1 infection and 717 of HTLV-2 infection had been recorded at the national Spanish HTLV registry [13].

Several serological surveys conducted over the last decade have monitored the prevalence of HTLV infections in Spain [14]. In the last serosurvey conducted in year 2008, a total of 7 out of 5,742 consecutive hospital outpatients were found to be HTLV-seroreactive (overall rate, 0.12%). No single case of HTLV-1 infection was reported at that time [15].

Recent estimates have pointed out that the number of immigrants legally registered as living in Spain has increased more than six-fold over the last 10 years [16]. Immigrants currently represent nearly 6 out of 47 million people in Spain. Many of the foreigners come from regions where the presence of HTLV-1 is endemic as some countries in Latin-America and Sub-Saharan Africa and where HTLV-3 and HTLV-4 have been reported occasionally [8]. This wave of immigration could have modified the prevalence of HTLV infection in Spain and the type distribution. In order to test this hypothesis, the Spanish HTLV Group conducted a new prospective, multicenter, national serosurvey for studying HTLV infection in year 2009.

## Results

A total of 6,460 consecutive adult outpatients attended during June 2009 at 16 distinct hospitals were screened for HTLV antibodies. The median age of the study population was 38 years and 40% were male. Although native Spaniards represented 88% of the total study population, 4.9% of subjects come from Latin America, 3.6% from Africa and 2.8% from other European countries.

Nine specimens were repeatedly EIA reactive and were confirmed by Western blot as HTLV-1 positive (n = 4) and HTLV-2 (n = 5). Another two samples exhibited EIA reactivity close to the cut-off but could not be confirmed as HTLV positive by Western blot or PCR testing. No PBMCs could be obtained from the four HTLV-1 positive cases, which precluded further virological characterization of these samples.

The prevalence of HTLV found in this study was 0.14%, being 0.06% for HTLV-1 and 0.08% for HTLV-2. Neither HTLV-3 nor HTLV-4 were found in this survey. Three of the HTLV-1 carriers had been born in Latin America (Peru, Ecuador and the Dominican Republic) while the last one was a 31 years-old native Spanish woman who denied any significant risk behavior for HTLV exposure, including intravenous drug use, sexual promiscuity, transfusions or stages in Latin America. All HTLV-1 carriers but one were asymptomatic. The woman from the Dominican Republic was newly diagnosed with mild TSP/HAM (Table 1).

**Table 1:** Main characteristics of HTLV EIA reactive individuals

Patient **No**.	HTLV type	Age (years)	Gender	Country of origin	Risk group	HTLV-associated illness	HIV
1	1	31	female	Spain	heterosexual	none	negative

2	1	60	female	Peru	transfusion	TSP/HAM	negative

3	1	41	female	Dominican Republic	heterosexual	none	negative

4	1	36	male	Ecuador	homosexual	none	positive

5	2	51	male	Spain	intravenous drug user	none	positive

6	2	41	male	Spain	intravenous drug user	none	positive

7	2	42	male	Spain	intravenous drug user	none	positive

8	2	47	female	Spain	intravenous drug user	none	positive

9	2	41	male	Spain	intravenous drug user	none	positive

10*	No	44	female	Venezuela	heterosexual	none	negative

11*	No	37	female	Spain	heterosexual	none	positive

*Indeterminate Western blot pattern

In contrast to HTLV-1 cases, all 5 subjects with HTLV-2 infection identified in this study were native Spaniards who admitted prior intravenous drug use. Moreover, all were coinfected with HIV. None of them complained of neurological symptoms potentially associated with HTLV-2 (Table 1).

The prevalence of HTLV infection in the current study seems to remain fairly stable (Table 2). Moreover, no cases of HTLV-3 nor HTLV-4 have been identified so far in Spain.

**Table 2:** Main characteristics of the two last cross-sectional surveys of HTLV antibodies conducted in Spain

Survey Year	2008^15^ (n = 5,742)	2009 (n = 6,460)	p
Male gender (%)	41.2	40	

Median age, years (IQR)	40 (31-57)	38 (29-53)	

Origin (%)			
Spain	92	88	
Latin America	3.4	4.9	
Africa	1.9	3.6	
Asia	0.2	0.6	
Other European countries	2.5	2.8	

**HTLV (n, %)**	7 (0.12)	9 (0.14)	0.9
HTLV-1	0 (0)	4 (0.06)	0.08
HTLV-2	7 (0.12)	5 (0.08)	0.62

## Discussion

For several years, periodic surveillance studies have been conducted in Spain looking for changes in the rate and distribution of HTLV infections. Overall the seroprevalence of HTLV infection found in this study was low (0.14%) and similar to that found in previous serosurveys carried out in Spain [14,15]. Only 9 out of 6,460 tested individuals were found to be positive for HTLV. While HTLV-2 seems to have been present for decades among native Spanish intravenous drug users [17], HTLV-1 seems to have been introduced more recently. The four cases identified in the current study support that the recent big wave of immigration from HTLV-1 endemic regions in Latin America and Africa, could have contributed to introduce HTLV-1 infection in Spain. Moreover, the recognition of HTLV-1 in a native Spaniard with lack of any evident risk for HTLV-1 exposure might further support that HTLV-1 is already spreading within the native Spanish population. In other European countries, as the United Kingdom and France, with larger and longer presence of immigrants from HTLV-1 endemic regions, sexual transmission of HTLV-1 from foreigners to natives has already been well documented [18].

The recognition of HTLV-1 infected persons in Spain has several clinical and public health implications. HTLV testing should be considered for a broader number of persons and conditions, including blood bank donors, organ transplantation, antenatal testing or sexually transmitted diseases clinics. Close relatives of infected individuals should be offered for HTLV testing. Given that most HTLV-1 carriers would remain asymptomatic life long, unaware silent transmission of the virus is the most worrisome. Wider HTLV testing may allow identification of carriers and help to reduce further transmission. Pregnant women with HTLV-1 infection should be advised against breast-feeding, which is the most effective way to prevent vertical HTLV-1 transmission. Moreover, physicians must monitor periodically asymptomatic persons known to be infected with HTLV-1, in order to facilitate early recognition of classical complications, such as TSP/HAM or ATL. Attention to mild symptoms or laboratory abnormalities, as in one of the current cases complaining minimal paraparesis, may permit early diagnosis and better treatment options.

The rate of HTLV-2 infection seems to remain fairly stable in Spain and confined mainly to former intravenous drug users and their sexual partners. As most HTLV-2 carriers are intravenous drug users coinfected with HIV and intravenous drug use practices have dramatically declined in Spain [19], we should expect a steadily decline in the prevalence of HTLV-2 infection in coming years.

It is worth to note that this is the largest study carried out in Spain examining the prevalence of HTLV infections. We are confident that the 16 hospitals that participated in the study properly covered the whole country. However, testing was made on hospital outpatients and therefore bias exists in terms of extrapolation of prevalence rates to the entire population. Studies conducted in other populations, including pregnant women, blood donors, immigrants, sexually transmitted disease clinics, would help to define more accurately the extent of HTLV infections in Spain. Of note, despite testing a relatively large immigrant population from Western Africa, where HTLV-3 and HTLV-4 were described originally [5-7] no single case of infection with these HTLV variants has been reported so far in Spain. In this study, the two samples from individuals showing weak EIA reactivity and indeterminate western blot results were negative for HTLV-3 and HTLV-4 testing. Moreover, they had no epidemiological link with Africa, being these two women from Venezuela and Spain, respectively. These results are in agreement with those from the United States, where a recent report has failed to identify HTLV-3 and/or HTLV-4 infection in risk groups, including individuals harboring indeterminate HTLV western blot patterns [20]. Although all individuals reported to date as infected with HTLV-3 or HTLV-4 have displayed reactivity on EIA designed for HTLV-1 and HTLV-2 screening [5,21], we should acknowledge that the overall sensitivity of these tests to pick up all HTLV-3 and/or HTLV-4 antibodies is currently unknown [22]. Thus, misdiagnosis of HTLV-3 and/or HTLV-4 in the present study might have occurred.

## Conclusions

This study supports that the prevalence of HTLV infection in Spain remains stable and low. However there is a slightly trend towards an increase in HTLV-1 mainly driven by the large immigration from endemic regions in Latin America over the last decades. The dramatic decline in intravenous drug use practices in recent years may result in a steadily decline in HTLV-2 in the near future. Altogether, HTLV-1 must be expected to take over HTLV-2 in coming years. Given that HTLV-1 is more pathogenic and that transmission often occurs from people unaware of their infection, HTLV testing should be considered for a broader number of persons.

## Methods

All adult outpatients attending the hospitals belonging to the HTLV Spanish Network, were invited consecutively during June 2009 to be tested for HTLV antibodies. In order to make sampling representative, the hospitals selected were distributed geographically across Spain and each of them recruited at least 350 samples. Every participating centre obtained approval from the corresponding ethical committee and informed consent was obtained from all recruited individuals.

Pools of five sera were screened for HTLV antibodies using a commercial enzyme immunoassay (EIA) (Murex HTLV I + II, Abbott, Madrid, Spain). This strategy has been evaluated previously and considered as appropriated for HTLV antibody testing [23]. Sera from reactive pools were re-tested individually by EIA and confirmed using a commercial Western blot (Genelabs Diagnostics, Redwood City, CA). Band patterns were interpreted following the HERN criteria [24]. Briefly, HTLV-1 or HTLV-2 positivity was considered when reactivity to at least two recombinant envelope bands (rgp21 and rgp46-I or II, respectively) and the gag band p24 were present. In samples yielding indeterminate Western blot pattern, when possible a new blood sample was drawn and peripheral blood mononuclear cells were obtained. Then, genetic confirmation discarded or confirmed HTLV infections using specific primers for HTLV types 1-4. Briefly, DNA was extracted from PBMCs with the midi spin columns from the QIAamp DNA blood Midi kit (Qiagen), using the procedure recommended by the manufacturer. Afterwards, polymerase chain reactions (PCR) were carried out on genomic *LTR *and/or *pol *regions, using primers and conditions described elsewhere [17,18,25]. The presence of amplicons was checked with electrophoresis on agarose gels. Finally, medical records were retrospectively reviewed for all individuals found to be HTLV positives.

### Statistical analysis

Results are given as proportions and median values. Comparisons were made using the chi square test, with Fisher correction when appropriated. Differences were considered to be significant only when p values were lower than 0.05. All analyses were performed using SPSS version 11.0 (SPSS Inc., Chicago, IL).

## HTLV Spanish study group

C. Rodríguez and J. del Romero (Centro Sanitario Sandoval, Madrid); C. Tuset, G. Marcaida and T. Tuset (Hospital General Universitario, Valencia); E. Caballero and I. Molina (Hospital Vall d'Hebron, Barcelona); A. Aguilera, J.J. Rodríguez-Calviño, S. Cortizo and B. Regueiro (Hospital Conxo-CHUS, Santiago); R. Benito and M. Borrás (Hospital Clínico Universitario Lozano Blesa, Zaragoza); R. Ortiz de Lejarazu and J.M. Eiros (Hospital Clínico Universitario, Valladolid); J.M. Miró, C. Manzardo, M.M. Gutiérrez and T. Pumarola (Hospital Clínic-IDIBAPS, Barcelona); J. García and I. Paz (Hospital Cristal-Piñor, Orense); E. Calderón, F.J. Medrano and M. Leal (Hospital Virgen del Rocío, Sevilla; CIBER de Epidemiología y Salud Pública); F. Capote (Hospital Puerta del Mar, Cádiz); A. Vallejo, F. Dronda and S. Moreno (Hospital Ramón y Cajal, Madrid); D. Escudero (Hospital Germans Trias i Pujol, Barcelona); E. Pujol (Hospital Juan Ramón Jiménez, Huelva); M. Trigo, J. Diz, P. Álvarez and M. García-Campello (Complejo Hospitalario, Pontevedra); M. Rodríguez-Iglesias (Hospital Universitario Puerta del Mar, Cádiz); A.M. Martín and A. Hernandez-Betancor (Hospital Universitario Insular, Las Palmas de Gran Canaria); J.M. Ramos, J. C. Rodríguez and F. Gutiérrez (Hospital General, Elche); C. Gómez-Hernando (Complejo Hospitalario Virgen de la Salud, Toledo); A. Guelar (Hospital del Mar, Barcelona); G. Cilla and E. Pérez-Trallero (Hospital Donostia, San Sebastián); J. López-Aldeguer (Hospital La Fe, Valencia); J. Sola (Hospital de Navarra, Pamplona); L. Fernández-Pereira (Hospital San Pedro de Alcántara, Cáceres); J. Niubó (Ciudad Sanitaria de Bellvitge, Barcelona); S. Veloso (Hospital Universitario, Tarragona); J.L. Gómez Sirvent, L. Arroyo, M.M. Alonso, M.R. Alemán and D. García Rosado (Hospital Universitario de Canarias, Santa Cruz de Tenerife); L. Force (Hospital General, Mataró); C. Cifuentes (Hospital Son Llatzer, Palma de Mallorca); J. García (Hospital de León); S. Pérez (Hospital do Meixoeiro, Vigo); C. Raya (Hospital del Bierzo, Ponferrada); A. González-Praetorius (Hospital Universitario, Guadalajara); A. Mena, J.L. Pérez and M. Peñaranda (Hospital Son Dureta, Mallorca); J.M. Montejo (Hospital de Cruces, Bilbao); N. Margall, M. Gutiérrez, P. Domingo (Hospital de Sant Pau, Barcelona); L. Roc and A. Martinez Sapiña (Hospital Miguel Servet, Zaragoza); I. Viciana (Hospital Virgen de la Victoria, Málaga); T. Cabezas, A. B. Lozano and J.M. Fernandez (Hospital de Poniente, Almería); I. García and G. Gaspar (Hospital Universitario de Getafe, Madrid); R. García and M. Gorgolas (Fundación Jiménez Díaz, Madrid); A. Treviño, P. Parra, C. de Mendoza and V. Soriano (Hospital Carlos III, Madrid).

## Competing interests

The authors declare that they have no competing interests.

## Authors' contributions

HTLV Spanish Study Group designed and conceived the study. AT, AA, EC, RB, JME, AH, EC, MR, AT, JG, JMR, LR, GM, CR, MT, CG, ROL and VS collected and provided data. AT, PP and CM analyzed the data. AT, CM, VS wrote the paper. All authors read and approved the final manuscript.
